# Intrinsically photosensitive retinal ganglion cells: Context-Dependent resilience and vulnerability

**DOI:** 10.1016/j.nbscr.2026.100153

**Published:** 2026-10-12

**Authors:** Arianna Cella, Ludovic S. Mure

**Affiliations:** a Inselspital, Department of Ophthalmology, Bern, Switzerland; b University of Bern, DBMR, Bern, Switzerland; c Inselspital, Zentrum für Experimentelle Neurologie, Department of Neurology, Bern, Switzerland

## Abstract

Retinal ganglion cells (RGCs) exhibit marked heterogeneity in their susceptibility to injury and disease. Intrinsically photosensitive retinal ganglion cells (ipRGCs), a small subset of melanopsin-expressing RGCs involved in non-image-forming visual functions, have emerged as a particularly informative example. In multiple experimental models, ipRGCs show relative resistance to acute insults such as optic nerve injury, excitotoxicity, and mitochondrial dysfuinction. In contrast, functional alterations and degeneration have been reported in aging and in neurodegenerative conditions including Alzheimer's and Parkinson's disease.

This apparent discrepancy suggests that ipRGC survival may depend on the nature and duration of the underlying stress. In this review, we summarize current evidence on ipRGC resilience and vulnerability across a range of experimental and pathological contexts. We discuss candidate mechanisms that may contribute to these responses, including sustained activity, calcium handling, metabolic specialization, neurochemical interactions, and gene expression programs. We also consider the role of subtype-specific diversity within the ipRGC population in shaping these outcomes.

Finally, we examine the potential of ipRGCs as biomarkers of disease and as a source of insight for neuroprotective strategies. Together, these observations support the view that ipRGC behaviour reflects an interaction between intrinsic cellular properties and the context in which they are engaged.

## Highlights

• ipRGCs show remarkable resilience to acute injury but vulnerability in chronic disease.
• ipRGC resilience and vulnerability vary with cell subtype and pathological context.
• Sustained activity and metabolic specialization may have context-dependent effects on ipRGC survival.
• ipRGC dysfunction may provide biomarkers and therapeutic targets in neurodegenerative disease.

## 1 Introduction

Retinal ganglion cells (RGCs) are the output neurons of the retina, responsible for transmitting visual information to the brain. These neurons are particularly vulnerable to a wide range of neurodegenerative insults, including glaucoma, optic neuritis, ischemia, and traumatic injury. Disorders involving RGC degeneration affect over 200 million individuals worldwide across all etiologies (Tham et al., 2014) and frequently result in irreversible vision loss and disability (Levin and Gordon, 2002). Despite their clinical importance, effective neuroprotective strategies for RGCs remain limited.

Importantly, RGC vulnerability is not uniform. Survival can vary by nearly two orders of magnitude across RGC types following injury such as optic nerve crush (Tran et al., 2019), indicating that intrinsic cellular properties strongly influence susceptibility to degeneration. Among these populations, intrinsically photosensitive retinal ganglion cells (ipRGCs) have emerged as a particularly intriguing case. In several experimental and pathological contexts, ipRGCs display a remarkable degree of resilience (Cui et al., 2015; Georg et al., 2017), yet in others, especially neurodegenerative diseases such as Alzheimer's and Parkinson's disease, they exhibit selective vulnerability (Morgia et al., 2016; Ortuño-Lizarán et al., 2018; Sanda et al., 2026).

ipRGCs are a small but functionally specialized subset of RGCs that express the photopigment melanopsin. Unlike conventional RGCs, which primarily mediate image-forming vision, ipRGCs are central to non-image-forming (NIF) visual functions, including circadian photoentrainment, pupillary light reflex, sleep regulation, and mood modulation. These functions are supported by projections to multiple brain regions, including the suprachiasmatic nucleus (SCN) and olivary pretectal nucleus (OPN). ipRGCs are themselves heterogeneous, comprising multiple subtypes (M1–M6) with distinct morphological, molecular, and functional properties (Aranda and Schmidt, 2020; Do, 2019; Mure, 2021; Sondereker et al., 2020).

The apparent paradox of ipRGC resilience in some contexts and vulnerability in others suggests that their survival is governed by context-dependent mechanisms, rather than a fixed intrinsic resistance. Understanding these mechanisms offers a unique opportunity to identify generalizable determinants of neuronal survival and to uncover novel therapeutic targets. In addition, because ipRGCs play a central role in regulating physiology and behaviour, their dysfunction may serve as a sensitive biomarker for disease onset and progression.

In this review, we synthesize current knowledge on the properties that distinguish ipRGCs from conventional RGCs in the context of stress and disease. We first provide an overview of ipRGC classification and physiology (Section 1). We then examine conditions in which ipRGCs display relative resistance (Section 2) or increased vulnerability (Section 3). In Section 4, we discuss candidate cellular and molecular mechanisms that may underlie these divergent responses, including intrinsic signaling pathways, metabolic adaptations, neurochemical interactions, and structural features. Finally, in Section 5, we explore how these insights can be leveraged for therapeutic intervention and biomarker development, and we outline key challenges and future directions.

## 2 ipRGCs are a functionally distinct class of RGCs

### 2.1 A new type of photoreceptor

ipRGCs represent a relatively recent addition to the catalog of retinal neuron types, first described in the early 2000s in the context of NIF visual functions. Observations in patients with outer retinal degeneration and in rodent models lacking rods and cones revealed preserved light-dependent physiological responses, such as circadian entrainment and pupillary constriction, despite severe loss of classical photoreceptors ((Foster et al., 2020) for review). These findings led to the identification of a distinct class of retinal ganglion cells expressing melanopsin, a photopigment encoded by the Opn4 gene.

ipRGCs constitute a small fraction of the total RGC population. Early estimates suggested approximately 2% in mouse retina (Berson et al., 2002, 2010, 2002; Hattar et al., 2002, 2006) and less than 1% in human retina (Hannibal et al., 2017); however, these values likely underestimate the full ipRGC population. In mice, several estimates preceded the identification of the low-melanopsin-expressing M4–M6 subtypes, while human estimates have largely relied on melanopsin immunohistochemistry, which may similarly underrepresent weakly expressing ipRGC populations (Maloney et al., 2024). Even if they constitute a minority, they play a central role in mediating non-visual responses to light. These include synchronization of the circadian clock to the external light-dark cycle, regulation of sleep and alertness, modulation of mood, and control of the pupillary light reflex. Genetic ablation or functional disruption of ipRGCs or melanopsin severely impairs these processes, indicating that ipRGCs serve as the primary, and in some cases exclusive, conduit for these responses (Altimus et al., 2008; Güler et al., 2008; Hatori et al., 2008; Mure et al., 2016).

ipRGCs exhibit characteristic photoresponses that distinguish them from conventional RGCs. Whereas most RGCs respond transiently to light onset or offset, ipRGCs display sustained responses that persist throughout light exposure and can continue for several seconds after stimulus offset (Berson et al., 2002; Dacey et al., 2005; Hattar et al., 2002; Mure et al., 2019). While melanopsin-mediated response measured in isolated ipRGCs is typically sluggish, ipRGCs also receive synaptic input from rods and cones, accelerating the kinetics and extending their intensity and spectral ranges (Belenky et al., 2003; Berson et al., 2002; Dacey et al., 2005; Wong et al., 2007).

### 2.2 ipRGCs are diverse

Despite their low abundance, ipRGCs are highly heterogeneous. At least six subtypes (M1–M6) have been identified based on differences in dendritic stratification within the inner plexiform layer, levels of melanopsin expression, intrinsic photosensitivity, and central projection patterns (Ecker et al., 2010; Karnas et al., 2013; Kim et al., 2019; Quattrochi et al., 2019; Schmidt and Kofuji, 2009; Tu et al., 2005). Recent molecular subtyping using single-cell RNA sequencing has further refined these classifications, identifying genetic markers that help defining ipRGC clusters (Kiyama et al., 2026; Peng et al., 2019; Rheaume et al., 2018).

These subtypes exhibit distinct functional specializations. For example, M1 ipRGCs provide major input to the SCN and are critical for circadian photoentrainment, but they also target the OPN and together with M2, mediate pupillary light reflex. Other ipRGC populations contribute to mood regulation (vLGN, IGL, perihabenular regions), and aspects of pattern vision (Chen et al., 2011; Ecker et al., 2010; Fernandez et al., 2018; LeGates et al., 2012; Schmidt et al., 2014; Sonoda and Schmidt, 2016). This functional diversity suggests that different ipRGC subtypes operate across distinct irradiance ranges and temporal domains.

The heterogeneity of ipRGCs also has important implications for their response to stress and disease. Increasing evidence indicates that resilience and vulnerability are not uniform across subtypes, raising the possibility that subtype-specific properties underlie differential survival.

### 2.3 ipRGCs in humans

The study of ipRGCs in humans has progressed substantially over the past decade (Mure, 2021). Morphological identification of melanopsin-expressing cells has been achieved in *post-mortem* retinal tissue (Esquiva et al., 2017; Hannibal et al., 2017; Liao et al., 2016; Nasir-Ahmad et al., 2017), and functional studies have begun to characterize their responses to light *ex vivo* (Cowan et al., 2020; Mure et al., 2019; Reinhard and Münch, 2021; Sanda et al., 2026).

Several ipRGC subtypes appear to be conserved between rodents and primates (Esquiva et al., 2017; Hannibal et al., 2017; Liao et al., 2016; Liu et al., 2023), and transcriptomic analyses indicate that ipRGCs are among the most conserved retinal neuron types across species (Peng et al., 2019; Rheaume et al., 2018). This conservation underscores their translational relevance and supports their use as targets for diagnostic and therapeutic strategies.

Given their central role in regulating physiology and behaviour, as well as their evolutionary conservation, ipRGCs provide a unique entry point for understanding how specific neuronal populations respond to stress and degeneration.

## 3 ipRGC resilience to disorders and experimentally induced injuries

Early after their discovery, ipRGCs were noted for their unusual resistance to injury. Across a range of experimental and pathological contexts, including axotomy, glaucoma, mitochondrial dysfunction, and excitotoxicity, ipRGCs consistently exhibit enhanced survival compared to conventional RGCs (Fig. 1, Table 1). However, this resilience is not absolute and varies depending on the nature of the insult, its duration, and the ipRGC subtype considered. Below, we summarize the main contexts in which ipRGC resilience has been characterized.

**Fig. 1:**
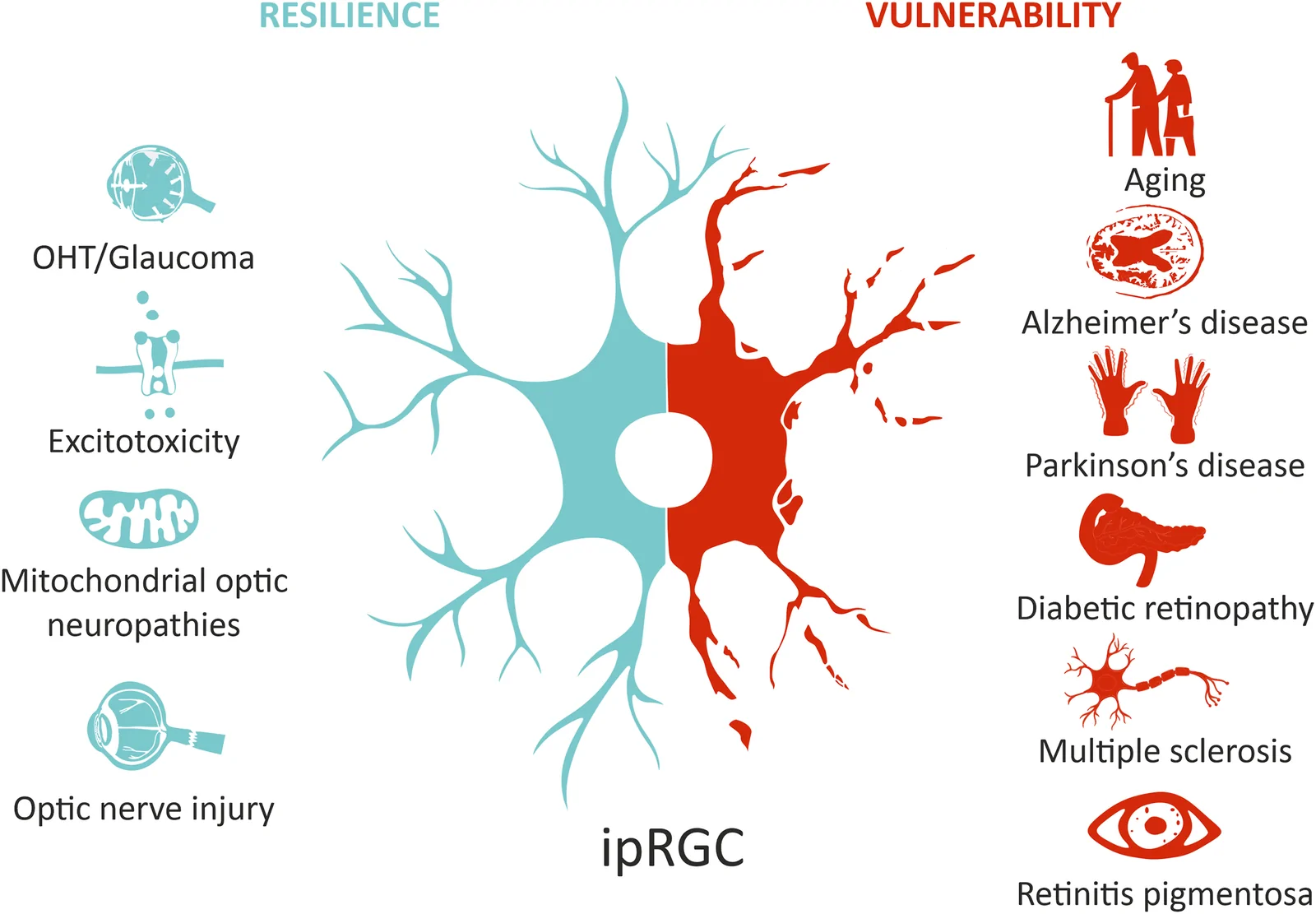
**The duality of ipRGCs pathophysiology.** Schematic representation of the responses of ipRGCs under different pathological environments.

**Table 1:** Experimental and clinical evidence of ipRGC response across injuries and diseases.

	Study	Species/Model	Detection Method	ipRGC Subtype(s)	Outcome
**Optic nerve injury (crush/transection*)***	Robinson and Madison (2004); Müller et al., 2014	Mouse/Rat, optic nerve transection	Melanopsin IHC	Not subtyped	**RESILIENT** — Higher ipRGCs survival rate compared to RGCs
	Tran et al. (2019); Jacobi et al. (2022); Tian et al. (2022)	Mouse, optic nerve crush	scRNA-seq	Multiple ipRGC clusters	**RESILIENT** — 5/7 most resilient molecular types are ipRGC-related; shared core survival/regeneration transcriptional programs
	Li et al. (2016)	Mouse, optic nerve injury; melanopsin KO/ectopic expression	Genetic manipulation + IHC + Behavior	M1–M3	**RESILIENT** — mTOR activity selectively retained in M1–M3
**Ocular hypertension/glaucoma**	Li et al. (2006)	Rat, laser OHT	Melanopsin IHC	Not subtyped	**RESILIENT** — preserved dendrites/survival
	Gao et al., 2022	Mouse, laser OHT	Subtype-specific reporter + Behavior	M1 vs. M4	**Mixed** — M1 spared (circadian intact); M4 vulnerable (contrast/acuity deficits)
	Jakobs et al., 2005	DBA/2J mouse	Melanopsin IHC	Not subtyped	**RESILIENT** — proportionally spared vs. >90% total RGC loss
	Kankipati et al., 2011; Feigl et al. (2011); Adhikari et al. (2016); Kelbsch et al. (2016); Najjar et al. (2018); Nissen et al. (2014); Denniss et al. (2025)	Human	Pupillometry	M1/M2	**Mixed** — intact/mild early, decline at later disease stage
	Obara et al., 2016	Human	Melanopsin IHC	Not subtyped	**VULNERABLE** — degeneration reported at later stages
***LHON/DOA***	La Morgia et al. (2010); Moura et al. (2013)	Human, LHON/DOA	Pupillometry + melatonin suppression	M1/M2	**RESILIENT** — function preserved
	Perganta et al. (2013)	Mouse, Opa1^Q285STOP	Melanopsin IHC + Pupillometry	M1	**RESILIENT** — morphology and function preserved
***Excitotoxicity***	Vidal-Villegas et al. (2019); Honda et al. (2019); Wang et al. (2018)	Mouse, NMDA	Melanopsin IHC	Not subtyped	**RESILIENT** — relative resistance
	DeParis et al. (2012)	Mouse, NMDA	Melanopsin IHC	Not subtyped	**RESILIENT** — resistance independent of PI3K/Akt, JAK/STAT
	Fogo et al. (2019)	Nile grass rat, NMDA	Melanopsin IHC + Behavior + Pupillometry	Not subtyped	**RESILIENT** — structure and function preserved
***Aging***	Esquiva et al. (2017)	Human, post-mortem	Melanopsin IHC	Multiple subtypes	**VULNERABLE** — decline in density/complexity after ∼ age 70
	Matynia et al. (2024)	Mouse	Melanopsin IHC + Pupillometry	M1	**RESILIENT** — morphology/function preserved into late adulthood
	Lax et al. (2016)	Rat	Melanopsin IHC	Not subtyped	**VULNERABLE** — stable 12–18 mo, ∼56% decline 18–24 mo
	Semo et al. (2003a), 2003b	Mouse, rd/rd cl	Melanopsin IHC + Pupillometry + Behavior	Not subtyped	**RESILIENT** — preserved despite photoreceptor loss and age >2 y
	Morgia et al., 2016	Human, post-mortem	Melanopsin IHC	Not subtyped	**VULNERABLE** — reduced density, dendritic atrophy
	Sanda et al., 2026	Human, post-mortem	MEA electrophysiology + IHC	Not subtyped	**VULNERABLE** — subtype-specific; hyperexcitability precedes loss
***AD***	Carrero et al. (2023)	Mouse, APP/PS1	Melanopsin IHC + CTB retrograde RHT tracing + ERG	Not subtyped	**VULNERABLE** — RHT/mRGC loss, smaller boutons, reduced ChAT
	Recio et al. (2026)	Mouse, 3xTg-AD	Melanopsin IHC + Behavior	M1	**Mixed** — early varicosity loss; PLR/CSF unchanged; sex- and quadrant-specific RGC loss
	Weigel et al. (2023)	Mouse, 3xTg-AD & 5xFAD	Melanopsin IHC + Behavior	Not subtyped	**Mixed** — ↑ density, ↑ light sensitivity (opposite of “loss”)
	Oh et al. (2019); Kawasaki et al. (2020)	Human, early AD	Pupillometry	M1/M2	**RESILIENT** — no alteration detected
	Romagnoli et al. (2020); Van Stavern et al. (2019)	Human	Pupillometry	M1/M2	**VULNERABLE** —alterations
	Asanad et al. (2021)	Human, preclinical AD	ERG	Inner retina	**VULNERABLE** — reduced PhNR before structural OCT change
***PD***	Ortuño-Lizarán et al. (2018)	Human, post-mortem	Melanopsin IHC	M1, M1d, M2	**VULNERABLE** — 25–30% density reduction, altered dendrites
	Joyce et al. (2018); Gaynes et al. (2022) Tabashum et al. (2021)	Human	Pupillometry	M1/M2	**VULNERABLE** — impaired; variable abnormality across patients
***HD***	Lin et al. (2019)	Mouse, R6/2 & N171-82Q	Melanopsin IHC + Pupillometry + Behavior	M1 vs. non-M1	**Mixed** — M1 lost (apoptotic); non-M1 relatively preserved
	Ouk et al. (2016)	Mouse, HD models	Pupillometry + Opsin expression	Not subtyped	**VULNERABLE** — attenuated PLR, ↓ opsin expression
***MS***	Meltzer et al. (2017)	Human	Pupillometry + OCT	M1/M2	**VULNERABLE** — diminished response
***Diabetes/DR***	Obara et al. (2017)	Human, post-mortem	Melanopsin IHC	Not subtyped	**VULNERABLE** — marked reduction
	Chen et al. (2021)	Mouse, streptozotocin-induced diabetes	Melanopsin IHC + Electrophysiology	M1 vs. M2/M3	**Mixed** — M1 structurally stable; M2/M3 remodeled, function altered
	Feigl et al. (2012)	Human, diabetes	Pupillometry	M1/M2	**VULNERABLE** — diminished pre-clinically
	Ishibashi et al., 2017	Human, type 2 diabetes	Pupillometry	M1/M2	**VULNERABLE** — impaired without autonomic neuropathy
***RP***	Esquiva et al. (2013)	Rat, P23H	Melanopsin IHC	Not subtyped	**VULNERABLE** — density decline up to 67% with age
	Lupi et al. (2012)	Mouse, rd/rd cl	Melanopsin IHC + Behavior	Not subtyped	**VULNERABLE** — loss reported with age
	Semo et al. (2003a), 2003b				**RESILIENT** — preserved (contrasts with Lupi et al., 2012)
***AMD***	Maynard et al. (2015), 2017	Human	Pupillometry	M1/M2	**VULNERABLE** — reduced constriction amplitude

### 3.1 Optic nerve injury (crush and transection models)

ipRGC resilience is best demonstrated in models of optic nerve injury. Following optic nerve transection or crush, ipRGCs, particularly the M1 subtype, are disproportionately preserved compared to other RGC populations. Early studies reported a threefold increase in survival of melanopsin-positive RGCs relative to non-melanopsin RGCs one month after transection (Robinson and Madison, 2004). Over time, this differential survival becomes even more pronounced, with ipRGCs constituting up to 82% of the remaining RGC population 60 days post-injury (Müller et al., 2014).

Large-scale transcriptomic analyses further support this observation. In a comprehensive classification of RGC types following optic nerve crush, five of the seven most resilient molecularly defined RGC populations were identified as ipRGCs or closely related clusters, indicating that resilience is a defining feature of this class (Tran et al., 2019). Subsequent studies have identified core transcriptional programs associated with survival and degeneration across RGC types (Jacobi et al., 2022; Tian et al., 2022), with ipRGCs prominently represented among the resistant populations.

Despite this robust survival, ipRGCs do not exhibit enhanced axonal regeneration compared to other RGCs(Robinson and Madison, 2004), indicating that the mechanisms governing survival and regeneration are at least partially dissociable.

### 3.2 Ocular hypertension and glaucoma

ipRGC resilience is also observed in models of ocular hypertension (OHT), which mimic aspects of glaucoma. In early studies using laser photocoagulation-induced OHT in rats, ipRGCs were reported to maintain dendritic morphology and exhibit relatively preserved survival compared to conventional RGCs (Li et al., 2006). However, more recent work indicates that this resilience is subtype-dependent. In a chronic OHT model induced by laser photocoagulation (Gao et al., 2022), M1 ipRGCs, primarily involved in circadian regulation via projections to the SCN, show minimal cell loss, and circadian behaviours such as re-entrainment remain largely intact. In contrast, M4 ipRGCs, which contribute to pattern vision, are significantly affected, with associated deficits in contrast sensitivity and visual acuity.

In genetic models of glaucoma, such as DBA/2J mice, melanopsin-positive cells are reduced in severely affected retinas (Jakobs et al., 2005). However, this reduction is proportionally much smaller than the overall loss of RGCs, which can exceed 90%. Thus, even when ipRGCs are affected, they remain relatively preserved compared to the broader RGC population.

Clinical observations in humans are broadly consistent with early resilience, although some studies suggest degeneration at later stages of disease (Obara et al., 2016). Functional assessments using pupillometry, including measurements of the post-illumination pupil response (PIPR), indicate that melanopsin-mediated responses can remain intact or only mildly impaired in early glaucoma (Adhikari et al., 2016; Denniss et al., 2025; Feigl et al., 2011; Kankipati et al., 2011; Kelbsch et al., 2016; Najjar et al., 2018; Nissen et al., 2014).

### 3.3 Mitochondrial optic neuropathies

Mitochondrial optic neuropathies, including Leber hereditary optic neuropathy (LHON) and dominant optic atrophy (DOA), provide a striking example of selective ipRGC preservation. These disorders are characterized by severe degeneration of conventional RGCs due to mitochondrial dysfunction, leading to profound visual impairment (Ng et al., 2021).

Remarkably, ipRGCs are largely spared in these conditions. In patients with advanced optic atrophy, ipRGC-dependent functions such as the pupillary light reflex and melatonin suppression remain relatively intact, indicating preservation of the retinohypothalamic pathway (La Morgia et al., 2010; Moura et al., 2013). Histological analyses and animal models support these findings, showing maintained ipRGC survival and morphology despite mitochondrial defects associated with mutations in genes such as OPA1 (Lenaers et al., 2021). OPA1 encodes a dynamin-related GTPase essential for mitochondrial structure, fusion, oxidative phosphorylation, calcium homeostasis, and apoptosis regulation (Lenaers et al., 2021). In mouse models carrying Opa1 mutations (e.g., B6; C3-Opa1Q285STOP), ipRGC morphology and function are preserved, further underscoring their resistance to mitochondrial dysfunction (Perganta et al., 2013).

These observations suggest that ipRGCs possess intrinsic properties that confer resilience to mitochondrial stress, although the precise mechanisms remain to be fully elucidated.

### 3.4 Excitotoxicity

ipRGCs also demonstrate significant resistance to excitotoxic injury. In models of NMDA-induced retinal damage, conventional RGCs undergo substantial degeneration, with losses reaching up to 85%, whereas ipRGCs show markedly greater survival (Vidal-Villegas et al., 2019; Wang et al., 2018). Although some studies report an initial reduction in melanopsin-positive cells shortly after insult (e.g., ∼25% loss at day 1), ipRGC numbers stabilize over time, suggesting relative long-term preservation (Vidal-Villegas et al., 2019). Consistent with these findings, Honda et al. (2019) reported that NMDA-induced loss of total RGCs reached approximately 58.4%, whereas melanopsin-positive ipRGC loss was limited to ∼7.6%, indicating a markedly higher tolerance of ipRGCs to excitotoxic stress. Importantly, this resistance appears to be independent of classical survival pathways such as PI3K/Akt and JAK/STAT signaling (DeParis et al., 2012), indicating that alternative protective mechanisms are involved.

Functional preservation is also observed. In a diurnal rodent model (Nile grass rat), NMDA-induced injury does not abolish ipRGC-mediated behaviours, including circadian photoentrainment, pupillary light reflex, and masking responses (Fogo et al., 2019). These findings indicate that ipRGCs not only survive excitotoxic insult but can also retain functional output.

### 3.5 Common features of ipRGC resilience across acute insults

Across these diverse models, optic nerve injury, ocular hypertension, mitochondrial dysfunction, and excitotoxicity, a consistent pattern emerges: ipRGCs exhibit enhanced survival relative to conventional RGCs.

Several shared features characterize these conditions. First, they typically involve acute or subacute stressors, such as axonal injury, excitotoxic signaling, or metabolic disruption, rather than prolonged degenerative processes. Second, the resilience of ipRGCs is frequently subtype-specific, with M1 cells often showing the highest degree of preservation. Third, functional outputs mediated by ipRGCs, such as circadian entrainment and pupillary responses, are often maintained even when substantial RGC loss has occurred.

However, this resilience is not uniform. Certain subtypes, such as M4 ipRGCs, are more vulnerable in specific contexts, and prolonged or severe insults can eventually affect ipRGC survival. These observations suggest that ipRGC resilience reflects a combination of intrinsic properties and contextual factors, setting the stage for the mechanistic analysis developed in subsequent sections.

## 4 ipRGCs are vulnerable to aging and neurodegenerative diseases

While ipRGCs exhibit resistance to a range of acute retinal injuries, a growing body of evidence indicates that they are susceptible to dysfunction and degeneration in the context of aging and chronic neurodegenerative diseases (Fig. 1, Table 1). This vulnerability is often accompanied by disturbances in NIF visual functions, including circadian rhythms, sleep regulation, and mood, which are frequently reported in these conditions. Importantly, these alterations often occur in parallel with broader retinal and central nervous system pathology, suggesting that ipRGC dysfunction may both reflect and contribute to disease progression. In contrast to acute injury models, where ipRGCs are relatively preserved, chronic conditions appear to engage mechanisms that progressively compromise their structure and function. It is important to note, however, that ipRGCs are not isolated when aging or degenerating. These conditions produce widespread changes in the retinal environment, including photoreceptor and interneuron dysfunction, synaptic remodeling, glial activation, inflammation, vascular and metabolic alterations, and changes in neurochemical signaling (Campello et al., 2021; Marc and Jones, 2003; Zhu et al., 2023). Consequently, alterations in ipRGC structure or function may reflect an interaction between cell-specific properties and changes in the retinal circuits and microenvironment in which these cells are embedded.

### 4.1 Aging

Aging is associated with structural and functional changes in ipRGCs, although the extent and timing of these alterations vary across species and studies. In humans, ipRGC density and dendritic complexity decline significantly after the age of 70, with evidence of reduced branching and morphological deterioration (Esquiva et al., 2017). Although these morphological changes occur in parallel with age-related alterations in sleep and circadian physiology, direct evidence linking the two is currently lacking. Age-dependent changes in ocular light transmission, other retinal circuits, and central circadian and sleep-regulatory structures are likely to contribute substantially to these physiological changes. In rodents, however, ipRGCs appear relatively preserved during mid-life. In mice, both morphology and pupillary light reflex (PLR) responses remain largely intact into late adulthood (Matynia et al., 2024), suggesting that ipRGC resilience may be maintained until advanced stages of aging.

Studies in Sprague–Dawley rats indicate a delayed but pronounced decline, with ipRGC density remaining stable at 12–18 months and decreasing by approximately 56% between 18 and 24 months (Lax et al., 2016). Interestingly, in models of severe outer retinal degeneration such as rd/rd cl mice, ipRGCs retain normal morphology and function even at advanced ages (>2 years), and both pupillary and circadian responses remain intact despite extensive photoreceptor loss (Semo et al., 2003a, 2003b).

Functionally, aging does not uniformly impair ipRGC-driven responses. While steady-state pupil constriction and melatonin suppression may remain relatively stable, more subtle changes have been reported, including shifts in spectral sensitivity and altered light input to central structures such as the suprachiasmatic nucleus (SCN) (Daneault et al., 2012; Lupi et al., 2012; Najjar et al., 2014). These changes may reflect a combination of intrinsic retinal remodeling and extrinsic factors such as lens yellowing and reduced light transmission. Notably, naturalistic studies indicate that melanopic irradiance remains a strong predictor of pupil behaviour across the lifespan, reinforcing the continued functional relevance of ipRGCs in aging individuals (Lazar et al., 2024).

Together, these findings suggest that ipRGCs are relatively resilient during early and mid-aging but become vulnerable at later stages, potentially as cumulative metabolic, structural, and environmental stresses exceed compensatory capacity.

### 4.2 Neurodegenerative diseases

Neurodegenerative disorders are frequently associated with non-motor symptoms such as sleep disruption, circadian misalignment, and mood disturbances. These symptoms are increasingly recognized as early and clinically significant features of disease, and their prevalence has prompted investigation into the role of ipRGCs and light signaling pathways. In some of these conditions, retinal pathology, including neuronal loss, thinning, protein aggregation, and inflammation, coexists with central neurodegeneration. Alterations in ipRGC structure and function may therefore arise both from local retinal changes and from systemic disease processes. Importantly, ipRGC dysfunction may not only serve as a biomarker but also contribute mechanistically to disease-associated circadian and behavioural disturbances.

#### 4.2.1 Alzheimer's disease

In Alzheimer's disease (AD), ipRGCs exhibit clear structural and functional alterations. *Post-mortem* analyses reveal reduced density, dendritic atrophy, and abnormal morphology of melanopsin-expressing cells, sometimes accompanied by local accumulation of amyloid-β deposits (Morgia et al., 2016; Sanda et al., 2026). Notably, La Morgia et al. reported melanopsin-positive RGC loss in AD in the absence of a corresponding reduction in conventional RGCs, supporting preferential vulnerability of the ipRGC population in this context. Functional changes are also reported, including increased excitability compared to age-matched controls, which may precede overt cell loss (Sanda et al., 2026).

Rodent models of AD have been used to probe this question directly, but the resulting picture is heterogeneous and, at times, contradictory. In the 3xTg-AD mouse, M1 ipRGC dendritic varicosities become smaller and less numerous as early as 4–8 months of age, prior to any measurable loss of conventional RGCs. This structural change is accompanied by an early- and late-onset light aversion phenotype without changes in contrast sensitivity or PLR amplitude, with the timing and pattern of RGC loss itself differing by sex and retinal quadrant (Recio et al., 2026). In the APP/PS1 model, Aβ deposition and reduced cholinergic tone in the retina are accompanied by a loss of melanopsin-positive RGCs, smaller axonal boutons, reduced retinohypothalamic tract projections to the SCN, downregulation of hypothalamic GABAergic signaling, and disrupted circadian clock gene rhythmicity in the hypothalamus and, to a lesser extent, hippocampus and cortex. Electroretinographic evidence confirm inner-retinal dysfunction (Carrero et al., 2023). Yet, paradoxically, behavioural studies in 3xTg-AD and 5xFAD mice have reported accelerated circadian re-entrainment after a simulated jet-lag, heightened negative masking responses to dim light, and a significant increase, not decrease, in ipRGC density. This suggests that, at least at some ages and in some models, ipRGC signaling may be transiently enhanced rather than diminished (Weigel et al., 2023), which would be consistent with the increased excitability observed in patients (Sanda et al., 2026). These discrepancies likely reflect differences in genetic background, sex, age, and the specific amyloid-versus tau-driven pathology of each model, and they underscore that the relationship between ipRGC structural integrity, functional output, and downstream circadian/behavioural consequences is neither linear nor fully understood.

Clinical functional assessments also present a more nuanced picture. Chromatic pupillometry has revealed altered post-illumination pupil responses consistent with melanopsin-pathway dysfunction in AD patients (Romagnoli et al., 2020) and subtle PLR abnormalities at the preclinical stage (Van Stavern et al., 2019), whereas other studies have failed to detect PLR deficits in pre-symptomatic individuals (Oh et al., 2019) or in early AD (Kawasaki et al., 2020). This apparent discrepancy suggests that ipRGC-driven pupillary deficits may only become reliably detectable once functional or structural loss has progressed beyond the earliest, pre-symptomatic stage of disease.

Importantly, these functional measures differ in their specificity for ipRGC dysfunction. The PIPR provides a relatively direct non-invasive readout of melanopsin-dependent retinal signaling, whereas circadian re-entrainment and masking reflect integrated responses involving ipRGC input together with downstream processing in central targets such as the SCN. Alterations in these behaviors therefore cannot, in isolation, be attributed specifically to retinal ipRGC dysfunction.

#### 4.2.2 Parkinson's disease

In Parkinson's disease (PD), ipRGC alterations have been documented primarily through *post-mortem* human studies. Immunohistochemical analyses reveal a reduction in ipRGC density, particularly affecting M1, M1d, and M2 subtypes, with decreases of approximately 25–30% compared to controls (Ortuño-Lizarán et al., 2018). Morphological analyses further demonstrate reduced dendritic complexity, including decreased branching and terminal points.

These changes may be linked to the degeneration of dopaminergic amacrine cells, which provide synaptic input to ipRGCs, particularly within the S1 stratum of the inner plexiform layer (Liao et al., 2016). Dopamine levels are reduced in the PD retina (Harnois and Di Paolo, 1990), and dopamine has been shown to regulate melanopsin and PACAP expression, potentially via D2 receptor-mediated mechanisms (Sakamoto et al., 2005). Loss of dopaminergic signaling may therefore impair ipRGC function and survival.

Functional assessments using pupillometry indicate abnormalities in melanopsin-mediated responses in PD patients (Gaynes et al., 2022; Joyce et al., 2018; Tabashum et al., 2021), although findings are variable and not entirely consistent across studies. Direct evidence from animal models remains limited, and some studies report no clear association between retinal α-synuclein pathology and ipRGC degeneration (Beach et al., 2014).

Taken together, these observations suggest that ipRGC vulnerability in PD may be driven, at least in part, by disruption of local dopaminergic circuits rather than intrinsic susceptibility alone.

#### 4.2.3 Huntington's disease

In Huntington's disease (HD), ipRGC dysfunction appears to occur early and may precede the onset of motor symptoms. In contrast to PD, ipRGCs show evidence of intrinsic, subtype-specific susceptibility. In the R6/2 mouse model, M1 ipRGCs are lost through apoptotic cell death, whereas non-M1 subtypes appear relatively preserved (Lin et al., 2019). Both R6/2 and N171-82Q mouse models exhibit impaired circadian rhythms, disrupted sleep patterns, and deficits in pupillary light responses (Lin et al., 2019; Ouk et al., 2016).

In humans, direct assessment of ipRGC-specific readouts such as the pupillary light reflex has not yet been reported in HD patients, but circadian and sleep-wake dysfunction consistent with impaired non-visual photoreception is well documented clinically. Patients show a delayed and blunted diurnal melatonin rise and reduced overall melatonin output (Aziz et al., 2009; Kalliolia et al., 2014), together with fragmented sleep, prolonged sleep latency and excessive daytime somnolence that can emerge even at the premanifest stage, well before motor onset (Goodman et al., 2011; Piano et al., 2015; Videnovic et al., 2014). Postmortem analysis of the human suprachiasmatic nucleus further reveals altered neuropeptide expression in HD patients (van Wamelen et al., 2013), raising the possibility that impaired central processing of retinal input, and not retinal dysfunction alone, contributes to these phenotypes. Together with the ipRGC and photopigment loss identified in mouse models (Lin et al., 2019; Ouk et al., 2016), these clinical observations suggest that ipRGC-dependent circuits may be compromised early in human HD as well, a hypothesis that remains to be tested directly with pupillometric or other ipRGC-specific measures.

Transcriptional dysregulation, a hallmark of HD pathology, is also observed in the retina and may contribute to ipRGC dysfunction (Cano-Cano et al., 2024). Interestingly, the mechanisms underlying ipRGC vulnerability in HD may involve mitochondria. La Morgia and colleagues (La Morgia et al., 2017) proposed that the increased mitochondrial fission characteristic of HD parallels the fusion defects caused by OPA1 mutations in dominant optic atrophy, pointing to a shared mitochondrial-dynamics vulnerability across these disorders, alongside potential contributions from transcriptional or proteostatic stress.

#### 4.2.4 Multiple sclerosis

In multiple sclerosis (MS), alterations in light perception and ipRGC-dependent responses have been reported, although the underlying mechanisms remain less well characterized. Patients frequently experience excessive daytime sleepiness and fatigue (Sguigna et al., 2023), as well as increased photosensitivity and photophobia (Cortese et al., 2018). Notably, these symptoms do not appear to correlate directly with clinical disability or prior episodes of optic neuritis, suggesting that they may arise from more subtle or diffuse alterations in retinal or central pathways. While direct evidence of ipRGC degeneration in MS is limited (Meltzer et al., 2017), the functional phenotype is consistent with impaired light signaling.

### 4.3 Metabolic and retinal disorders

#### 4.3.1 Diabetes and diabetic retinopathy

In diabetes, ipRGCs exhibit subtype-specific structural and functional alterations. Histological analyses of human retinas with advanced diabetic retinopathy reveal a marked reduction in melanopsin-positive cells (Obara et al., 2017), while animal models show more nuanced changes.

In streptozotocin-induced diabetic mice, M1 ipRGCs remain structurally and functionally stable even after prolonged hyperglycemia (3–6 months), whereas M2 and M3 subtypes undergo significant remodeling, including increased soma size and dendritic complexity (Chen et al., 2021). Despite preserved melanopsin expression, functional responses in these cells are altered, with changes in response gain and impaired PLR.

More broadly, ipRGC morphology and function are affected in diabetic models even in the absence of significant cell loss. Furthermore, STZ diabetic mice show reduced circadian sensitivity to low light intensities, and delayed re-entrainment after a phase advance (Gastinger et al., 2008; Kumar and Zhuo, 2011; Lahouaoui et al., 2014). Clinical studies further indicate that melanopsin-mediated pupil responses are diminished in diabetic patients without overt retinopathy, suggesting early subclinical dysfunction (Feigl et al., 2012; Ishibashi et al., 2017). These findings indicate that ipRGC dysfunction can occur early in diabetes and may provide a retinal marker of disease. Whether these alterations contribute causally to the circadian disturbances associated with diabetes remains to be established. Finally, ipRGCs also contribute to the regulation of glucose metabolism. In mice, light exposure reduces glucose tolerance through ipRGCs that project to the supraoptic nucleus (SON), independently of the SCN(Meng et al., 2023). These SON-projecting cells form a different subset of M1 ipRGCs located in the dorsal retina (Berry et al., 2023). Together with the relative stability of M1 cells in diabetic mice described above, this could suggest that the ipRGCs regulating glucose metabolism and those most affected by hyperglycemia may be distinct populations.

#### 4.3.2 Inherited retinal degenerative diseases

In retinal degenerative conditions such as retinitis pigmentosa (RP), ipRGCs undergo progressive structural and functional alterations, although findings vary across models. In the P23H rat model, ipRGC density declines by up to 67% with age, accompanied by reduced dendritic complexity (Esquiva et al., 2013). Similarly, in rd/rd cl mice, ipRGC loss has been reported following photoreceptor degeneration, suggesting transneuronal degeneration (Lupi et al., 2012). However, other studies indicate preserved ipRGC morphology and function even in the context of severe outer retinal degeneration: rodless/coneless mice retain circadian and pupillary responses into old age (Semo et al., 2003a, 2003b), and patients with RP, including those with a non-recordable ERG, maintain PIPR to bright blue light (Kardon et al., 2011). These discrepancies that may reflect differences in disease stage, model systems, or methodological approaches. Progressive remodeling of retinal circuits is likely to play a key role in shaping ipRGC outcomes in these conditions (Eleftheriou et al., 2020; Jones and Marc, 2005; Marc and Jones, 2003).

#### 4.3.3 Age-related macular degeneration

Direct evidence of ipRGC degeneration in age-related macular degeneration (AMD) remains limited and, to our knowledge, the relative loss of ipRGCs compared with other RGC populations has not been sufficiently characterized to establish preferential vulnerability. However, several indirect observations suggest potential involvement. AMD patients frequently report sleep disturbances, altered circadian rhythms, and a high prevalence of depression (Casten and Rovner, 2008; Khurana et al., 2016; Maynard et al., 2017; Pérez-Canales et al., 2016; Rees et al., 2010). In addition, pupillary light responses are often impaired, with reduced constriction amplitude (Feigl and Zele, 2014; Maynard et al., 2015, 2017). While these changes may be influenced by visual impairment and reduced light exposure, they are also consistent with altered ipRGC function. The contribution of ipRGC dysfunction to mood disturbances in AMD remains an open question but is particularly relevant given the known role of ipRGC pathways in affective regulation (Fernandez et al., 2018).

### 4.4 Synthesis: selective vulnerability in chronic conditions

Across aging, neurodegenerative, and metabolic disorders, a consistent pattern emerges: ipRGCs are not uniformly resistant but instead exhibit context- and subtype-dependent vulnerability. In many cases, functional alterations precede overt cell loss, suggesting that early dysfunction of light signaling pathways may contribute to disease symptoms.

Mechanistically, these conditions are characterized by chronic stressors, including mitochondrial dysfunction, oxidative stress, protein aggregation, inflammation, and loss of neurochemical support, that differ fundamentally from the acute insults in which ipRGCs are resilient. These stressors are likely to interact with the intrinsic properties of ipRGCs, such as sustained activity, high calcium flux, and elevated metabolic demand, and could potentially shift the consequences of these features from protective to deleterious. This emerging picture raises the possibility that ipRGC resilience and vulnerability reflect context-dependent outcomes of overlapping biological properties operating under different temporal and pathological conditions.

## 5 Hypothetical mechanisms underlying ipRGC-specific resilience and vulnerability

ipRGCs display a striking duality in their response to stress: they are highly resistant to acute insults such as axotomy, excitotoxicity, and ocular hypertension, yet lose this resistance and can be affected in chronic conditions including aging and neurodegenerative diseases. Converging evidence suggests that this apparent paradox arises from a combination of intrinsic molecular, functional, and structural properties that confer context-dependent advantages. Importantly, several of these same features may become maladaptive under sustained or cumulative stress, providing a mechanistic basis for both resilience and vulnerability (Fig. 2). To clarify this duality, the mechanisms discussed below are organized into major functional axes that differentially contribute to survival depending on the nature and duration of the insult.

**Fig. 2:**
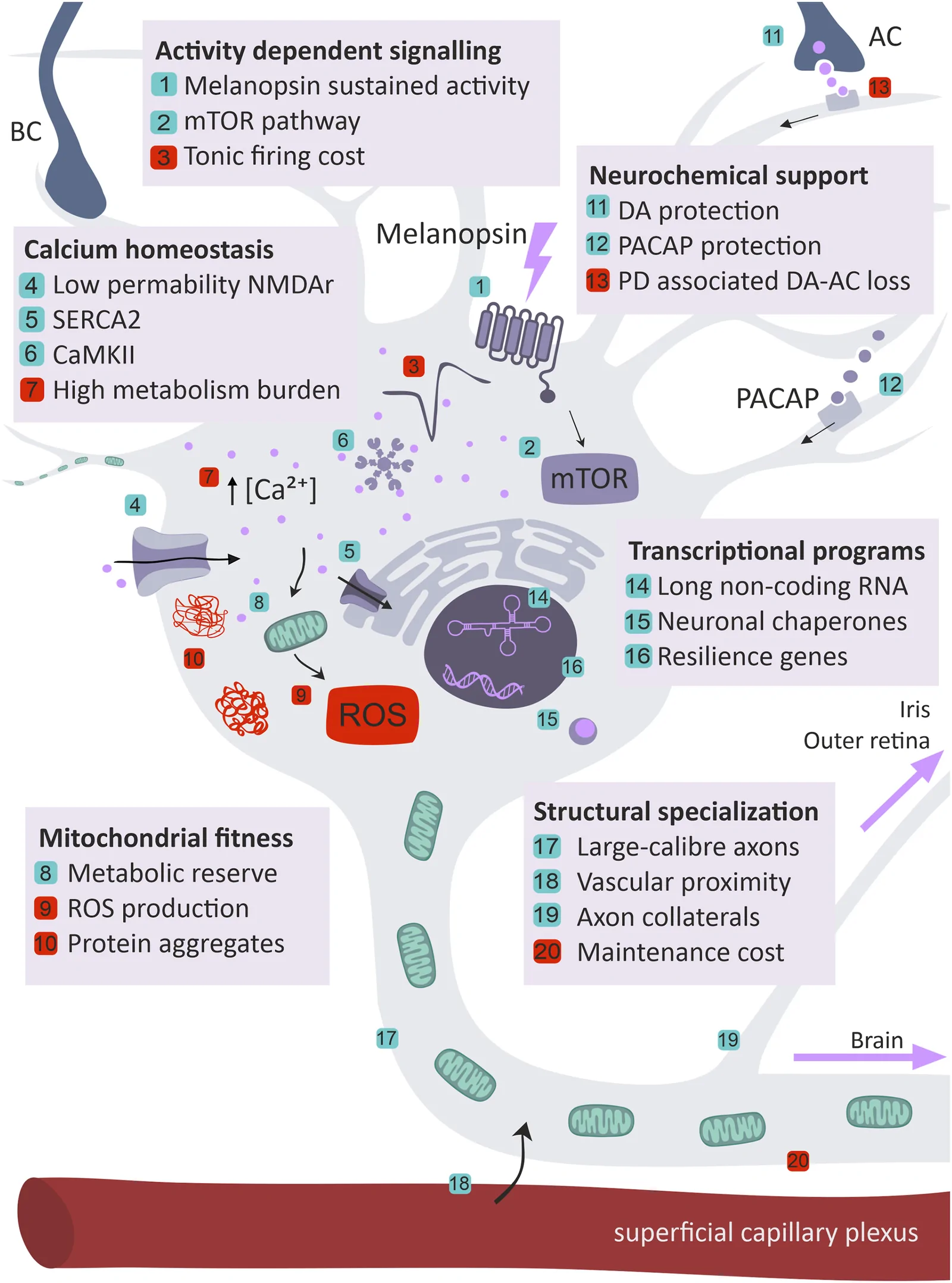
Putative mechanisms behind ipRGC context-specific resilience and vulnerability.

Two caveats need to be mentioned. First, the current evidence that ipRGCs are more vulnerable than other retinal neurons in chronic conditions, which also involve various retinal and brain dysfunctions, is limited. We hypothesised that protective characteristics under acute stress can become costly under chronic stress. Second, not all properties that distinguish ipRGCs are independent. Melanopsin signalling, calcium handling, mitochondrial density and gene expression are intrinsic to ipRGCs; neurochemical support and some structural advantages depend on other cell types or on the local environment and may be lost when these are affected by disease, as in Parkinson's disease with the dysfunction of dopaminergic amacrine cells. We therefore present this acute-to-chronic transition as a working framework rather than an established mechanism.

### 5.1 Activity-dependent protection and sustained signaling

A defining feature of ipRGCs is their sustained light-driven activity, which distinguishes them from the predominantly transient responses of conventional RGCs. This functional property appears closely linked to survival outcomes. Indeed, RGCs with sustained responses survive approximately threefold better than transient types following optic nerve injury (Tran et al., 2019). Consistently, five of the seven most resilient molecularly defined RGC types identified in this study were ipRGCs or closely related sustained αRGCs.

Melanopsin phototransduction underlies this sustained activity. Upon light activation, melanopsin engages a Gq/11-coupled cascade that activates phospholipase Cβ (PLCβ), leading to the hydrolysis of PIP_2_ into IP_3_ and DAG. IP_3_ mobilizes intracellular calcium stores, while DAG contributes to the activation of TRPC channels, resulting in prolonged depolarization and sustained calcium influx (Contreras et al., 2021; Do, 2019). This signaling mode contrasts sharply with the transient responses of rods and cones and imposes a distinct intracellular signaling regime.

Functionally, sustained activity has been linked to the maintenance of pro-survival pathways. In particular, melanopsin signaling promotes sustained activation of the mammalian target of rapamycin (mTOR) pathway following optic nerve injury. M1–M3 ipRGCs retain mTOR activity under these conditions, in contrast to αRGCs, and this effect is attenuated in melanopsin knockout models (Li et al., 2016). Ectopic expression of melanopsin in conventional RGCs is sufficient to enhance axonal regeneration via mTORC1 activation in a light- and Gq/11-dependent manner. Although this effect appears more closely related to regeneration than to survival per se, as it does not involve increased Akt phosphorylation (Li et al., 2016). This experimentally induced regenerative effect contrasts with the lack of enhanced spontaneous axonal regeneration reported for endogenous ipRGCs following injury (Robinson and Madison, 2004), indicating that melanopsin-dependent mTOR activation alone is insufficient to determine regenerative capacity.

Together, these findings suggest that sustained melanopsin-driven activity supports survival by maintaining trophic signaling pathways after acute injury. However, this same persistent activation may impose long-term metabolic and ionic burdens that become detrimental under chronic stress conditions.

### 5.2 Calcium homeostasis: buffering capacity versus chronic overload

Calcium (Ca^2+^) is a central regulator of neuronal function and survival, and its dysregulation is a well-established driver of neurodegeneration (McCracken and Williams, 2025). The signaling properties of ipRGCs can expose them to sustained calcium influx, although the magnitude and sources of this calcium load are likely to differ substantially across ipRGC subtypes according to their intrinsic excitability and firing properties (Hartwick et al., 2007; Hu et al., 2013; Milner and Do, 2017). Several features distinguish ipRGC calcium physiology from that of conventional RGCs. These include (i) expression of NMDA receptor subunits with reduced calcium permeability (Cui et al., 2015; Fogo et al., 2019), (ii) higher baseline intracellular Ca^2+^ levels that correlate with increased resilience (McCracken et al., 2023), and (iii) enhanced buffering and clearance mechanisms, potentially involving CaMKII-mediated signaling (Guo et al., 2021) and sustained activity of the sarco/endoplasmic reticulum Ca^2+^-ATPase SERCA2 (Shiga et al., 2024). CaMKII expression has been identified in a subset of primate RGCs, including melanopsin-expressing cells, and promotes survival following both excitotoxic and axonal injury (Baldicano et al., 2022; Guo et al., 2021).

These adaptations likely contribute to the relative resistance of ipRGCs to excitotoxic damage. However, calcium handling also represents a potential point of failure. Sustained calcium entry increases metabolic demand, as mitochondria must buffer cytosolic Ca^2+^ via the mitochondrial calcium uniporter, thereby stimulating oxidative phosphorylation to meet ATP requirements. Under pathological conditions, impaired calcium clearance can precede cell death. Indeed, recent work shows that injured RGCs exhibit slower Ca^2+^ clearance associated with endoplasmic reticulum stress and downregulation of SERCA2, a phenotype observed in models of ocular hypertension and optic nerve crush as well as in human glaucoma. Notably, AAV-mediated restoration of SERCA2 rescues calcium dynamics, reduces ER stress, and promotes neuronal survival (Shiga et al., 2024). Beyond its direct effects on excitability, disruption of ER calcium homeostasis is also expected to compromise proteostasis. Many ER-resident chaperones require high luminal Ca^2+^ concentrations to ensure proper protein folding, and depletion of ER calcium promotes activation of the unfolded protein response, impaired proteostasis, and accumulation of misfolded proteins. Thus, chronic disturbances in calcium handling may provide a direct mechanistic link between sustained neuronal activity, ER stress, mitochondrial dysfunction, and the progressive protein aggregation characteristic of many neurodegenerative diseases.

Thus, while enhanced calcium handling may protect ipRGCs during acute stress, chronic calcium load may contribute to progressive dysfunction and degeneration.

### 5.3 Metabolic and mitochondrial specialization: reserve capacity and long-term liability

ipRGCs exhibit features consistent with elevated metabolic capacity, including dense mitochondrial content within dendrites and strong cytochrome oxidase activity (Belenky et al., 2003; Georg et al., 2017; La Morgia et al., 2011; von Bussmann et al., 1993). This specialization is likely necessary to sustain prolonged depolarization and calcium influx associated with melanopsin signaling.

Enhanced mitochondrial content may confer a survival advantage in conditions characterized by mitochondrial dysfunction. In mitochondrial optic neuropathies such as LHON and DOA, ipRGCs are relatively spared, suggesting that increased mitochondrial mass and functional redundancy provide resilience. Potential mechanisms could include greater spare respiratory capacity, dilution of mutant mitochondrial DNA, and more effective mitochondrial quality control through biogenesis and PINK1/Parkin-mediated mitophagy (Lenaers et al., 2021).

However, this metabolic profile may also impose long-term costs. Sustained oxidative phosphorylation, combined with continuous calcium handling and axonal transport demands, is expected to increase reactive oxygen species (ROS) production and promote mitochondrial damage over time. Accumulation of oxidative stress, lipid peroxidation, and mitochondrial DNA mutations may progressively overwhelm cellular homeostasis, particularly in aging and neurodegenerative contexts such as Alzheimer's disease. Chronic mitochondrial dysfunction is also expected to impair proteostasis through ATP depletion, oxidative modification of proteins, reduced proteasomal activity and impaired autophagic flux, thereby promoting the accumulation of misfolded proteins characteristic of several neurodegenerative diseases.

This dual role of mitochondrial specialization provides a plausible explanation for the selective resilience of ipRGCs in acute mitochondrial insults and their vulnerability under chronic degenerative conditions.

### 5.4 Neurochemical support and local circuit interactions

ipRGCs occupy a distinctive neurochemical niche that may contribute to their resilience. A key feature is the expression of pituitary adenylate cyclase-activating polypeptide (PACAP), a neuropeptide with well-established neuroprotective properties in the retina (Hannibal et al., 2002, 2004, 2014; Patko et al., 2022). The retinoprotective effects of PACAP have been demonstrated across numerous models of retinal injury, including excitotoxicity, optic nerve injury, ocular hypertension, retinal ischemia, retinopathy of prematurity, diabetic retinopathy and age-related macular degeneration (Fabian et al., 2019; Kvarik et al., 2021; Maugeri et al., 2017, 2019; Seki et al., 2006, 2008, 2011; Shioda et al., 2016; Szabadfi et al., 2012). Moreover, PACAP-deficient mice exhibit increased retinal vulnerability and accelerated retinal aging (Kovács-Valasek et al., 2017; Kvarik et al., 2021; Szabadfi et al., 2012), supporting a physiological role for endogenous PACAP in maintaining retinal homeostasis. Because melanopsin-expressing ipRGCs constitutively express PACAP, it has long been proposed that this neuropeptide contributes to their intrinsic resistance to injury (La Morgia et al., 2011), although direct evidence that endogenous PACAP mediates the selective resilience of ipRGCs remains lacking.

ipRGCs are also closely associated with dopaminergic amacrine cells. M1 dendrites receive synaptic input in the S1 stratum of the inner plexiform layer, and ipRGCs express dopamine receptors such as D1a (Van Hook et al., 2012; Vugler et al., 2007). Dopamine acting through D1 receptors reduces the amplitude of melanopsin-driven photoresponses, thus modulating ipRGCs excitability and it has neuroprotective effects against glutamate-induced excitotoxicity (Müller et al., 2014), suggesting that local dopaminergic signaling may support ipRGC survival. Conversely, degeneration of dopaminergic neurons in Parkinson's disease may deprive ipRGCs of this support, potentially contributing to their vulnerability in this context.

Together, these findings suggest that ipRGC resilience is not purely cell-autonomous but may also depend on local neurochemical interactions that can be disrupted in disease.

### 5.5 Structural and anatomical features

ipRGCs exhibit several morphological characteristics that may influence their susceptibility to injury. They are among the largest RGCs, with large-caliber axons (Do, 2019; Kim et al., 2019), a feature associated with more efficient axonal transport and potentially greater resilience to metabolic stress (Perge et al., 2009; Yu et al., 2013). This may be particularly relevant in mitochondrial disorders, where larger axons could better tolerate impaired energy production (Lenaers et al., 2021; Pan et al., 2012; Wong et al., 2023). A downside of this large caliber, however, is the energetic cost of maintaining such structures, since larger axons require proportionally more mitochondria and generate correspondingly more metabolic byproducts (Perge et al., 2009; Yu et al., 2013), a cost that may only become significant under chronic bioenergetic stress.

In addition, a subset of M1 ipRGCs possesses intra-retinal axon collaterals that branch within the retina, iris, or ciliary marginal zone (Joo et al., 2013; Semo et al., 2014). Because these collaterals diverge before exiting the retina, they may retain access to local trophic support following optic nerve injury, potentially enhancing survival. However, this feature is limited to a small fraction of M1 cells and is unlikely to account for the broader resilience of the ipRGC population.

Finally, M1 dendrites are often located near the superficial capillary plexus, which may provide preferential access to metabolic substrates and protect against early vascular deficits, such as those observed in diabetic retinopathy (Chen et al., 2021). Notably, recent work suggests that vascular proximity can confer protection independently of mTOR signaling, highlighting the importance of microenvironmental factors (Nimkar et al., 2025).

Another hypothesis linked to melanopsin expression is that the photopigment could shield the cell by quenching light, especially short-wavelength light that would otherwise be absorbed by mitochondrial proteins and contribute to oxidative damage (Georg et al., 2017; Osborne et al., 2014), a form of intrinsic photoprotection that would not be available to non-photosensitive RGC types.

While these structural features may modulate vulnerability, they are unlikely to be sufficient on their own to explain the distinctive survival profile of ipRGCs.

### 5.6 Gene expression programs and stress response pathways

Transcriptomic analyses reveal that ipRGCs engage distinct gene expression programs in response to injury. These include differential expression of long non-coding RNAs (Ayupe et al., 2021) and microRNAs (Mead et al., 2021), suggesting specialized post-transcriptional regulation of stress responses.

ipRGCs also express high levels of the neuronal chaperone proSAAS, which inhibits aggregation of proteins such as α-synuclein and amyloid peptides and protects against oligomer-induced cytotoxicity. Transcriptomic datasets indicate that ipRGCs are among the highest expressers of proSAAS (Li et al., 2024; Tran et al., 2019), an observation that sits somewhat uneasily with the reported vulnerability of ipRGCs in several proteinopathies, including Alzheimer's and Parkinson's disease. However, immunohistochemical data do not clearly demonstrate preferential localization, raising questions about proSAAS functional contribution *in vivo*. Another possibility is that proSAAS confers protection against acute or short-term proteotoxic insults but is insufficient against the decades-long accumulation of amyloid-β or α-synuclein pathology characteristic of these diseases; another is that immunohistochemical detection of the protein does not necessarily reflect its functional, chaperone-active pool.

In addition, injury-induced upregulation of genes such as Thbs1, encoding thrombospondin-1, has been shown to promote axonal regeneration and may contribute to the distinct regenerative and survival responses of ipRGCs (Bray et al., 2019). Other factors, such as osteopontin (Spp1), are enriched in both αRGCs and ipRGCs and may participate in shared resilience pathways (Zhao et al., 2023).

These molecular programs likely interact with the functional and metabolic features described above to shape the overall response of ipRGCs to stress.

### 5.7 Synthesis: a context-dependent model of ipRGC resilience

Taken together, the survival advantage of ipRGCs appears to arise from a combination of sustained activity-dependent signaling, efficient calcium handling, enhanced metabolic capacity, neurochemical support, and specialized gene expression programs. These features collectively promote survival under acute stress conditions such as axonal injury or excitotoxicity.

However, the same properties, persistent activity, high calcium flux, and elevated metabolic demand, may render ipRGCs susceptible to cumulative damage over time, particularly in the context of aging and neurodegenerative disease. This hypothesis provides a testable framework for the dual behaviour of ipRGCs and suggests that resilience and vulnerability may represent context-dependent outcomes arising from interactions between cell-intrinsic properties and the surrounding retinal environment. The impact of these mechanisms is critically dependent on timescale: processes that support survival over hours to days (e.g., sustained activity or metabolic activation) may become deleterious over months to years.

## 6 Opportunities, translational perspectives, and remaining challenges

The distinctive properties of ipRGCs, particularly their context-dependent resilience and vulnerability, offer multiple opportunities for both therapeutic intervention and biomarker development. However, translating these insights into clinical applications requires addressing several conceptual and technical challenges.

### 6.1 Identifying therapeutic targets from resilience mechanisms

Advances in high-throughput transcriptomics, including single-cell RNA sequencing (scRNA-seq) and bulk RNA-seq of RGC-enriched samples, have enabled the construction of detailed molecular atlases of retinal ganglion cell types (Hahn et al., 2023). These approaches have identified multiple ipRGC subpopulations in both mouse and primate retinas and revealed distinct gene expression profiles associated with resilience and susceptibility.

Comparative analyses of RGC responses to injury have proven particularly informative. For example, scRNA-seq studies in optic nerve crush models have identified transcriptional programs associated with survival, including those enriched in ipRGCs (Jacobi et al., 2022; Tian et al., 2022; Tran et al., 2019). These datasets provide a foundation for identifying candidate pathways that could be targeted to enhance survival in vulnerable RGC populations.

Despite this progress, translation into therapeutic strategies remains limited. One challenge is that many identified pathways are context-dependent and may have different effects depending on the type and duration of injury. Another is that resilience mechanisms often involve complex, coordinated programs rather than single targets, complicating pharmacological intervention.

### 6.2 Developing ipRGC-based biomarkers

ipRGC function can be assessed non-invasively using pupillometry, making it an attractive candidate for biomarker development (Gamlin et al., 2007; Mure et al., 2009). The post-illumination pupil response (PIPR), which reflects melanopsin-driven activity, has been explored in a range of conditions, including Alzheimer's disease, Parkinson's disease, glaucoma, and diabetes (Feigl et al., 2011, 2012; Gaynes et al., 2022; Ishibashi et al., 2017; Kawasaki et al., 2020; Oh et al., 2019; Romagnoli et al., 2020; Tabashum et al., 2021; Van Stavern et al., 2019). However, results across studies have been variable. This variability likely reflects differences in experimental protocols, including light wavelength, intensity, duration, and temporal structure (Chougule et al., 2019; Romagnoli et al., 2023). In addition, the PLR primarily reflects the activity of M1 and M2 ipRGC subtypes (Baver et al., 2008), with M1 cells often being the most resilient. As a result, pupillometry may lack sensitivity to early or subtype-specific dysfunction. A key challenge is therefore to define the stimulus domains (wavelength × intensity × temporal profile) that best reveal disease-specific alterations in ipRGC function. Identifying such signatures could improve both sensitivity and specificity and enable differentiation between disorders with overlapping phenotypes.

Complementary approaches include electroretinography (ERG), particularly the photopic negative response (PhNR), which reflects inner retinal function. PhNR reductions have been reported in preclinical Alzheimer's disease, even in the absence of detectable structural changes on optical coherence tomography (Asanad et al., 2021). Combining pupillometry, ERG, actigraphy, and structural imaging may provide a more comprehensive assessment of ipRGC function and its contribution to disease.

### 6.3 Managing comorbidities through light-based interventions

Alterations in ipRGC-mediated light signaling can have significant consequences for circadian regulation, sleep, mood, and overall quality of life. In many disorders, these alterations may not only be a consequence of disease but also contribute to its progression. Light-based interventions offer a promising, low-cost, and scalable strategy to compensate for impaired ipRGC function. Optimizing light exposure, in terms of timing, intensity, and spectral composition, may improve circadian entrainment, sleep quality, and mood. Clinical studies have demonstrated beneficial effects of light therapy in conditions such as Parkinson's disease (Martino et al., 2018; Videnovic et al., 2017), although protocols remain heterogeneous and require further standardization. A similar rationale extends to Alzheimer's disease and related dementias, where trials of scheduled bright light exposure have improved rest–activity rhythms and reduced behavioural disturbances in older adults with dementia (Lek et al., 2008). A major challenge is to tailor interventions to the specific alterations in ipRGC function associated with each disorder, taking into account subtype-specific contributions and disease stage.

### 6.4 Translational challenges and open questions

Despite significant progress, several key questions remain unresolved. First, the extent to which resilience and vulnerability are subtype-specific remains incompletely understood. Many early studies relied on melanopsin immunostaining, which predominantly labels M1 ipRGCs, and may therefore not capture the full diversity of responses across subtypes (Table 1). Recent evidence suggests that different ipRGC subtypes exhibit distinct susceptibility profiles, but a comprehensive mapping is still lacking.

Second, the temporal dynamics of ipRGC dysfunction relative to disease progression are poorly characterized. Most studies compare healthy and diseased states at limited time points, making it difficult to determine whether ipRGC alterations are early events or downstream consequences.

Third, it remains unclear whether different diseases produce distinct “ipRGC phenotypes”. For example, reductions in PIPR amplitude are observed across multiple neurodegenerative conditions, raising the question of whether disease-specific signatures can be identified or whether ipRGC dysfunction converges onto common pathways.

Finally, an important and often overlooked factor is biological sex. Most studies of non-image-forming light responses have been conducted predominantly in male animals (Dib et al., 2021; Lee et al., 2021; Obodo et al., 2023; Spitschan et al., 2022). However, emerging evidence indicates sex differences in light sensitivity, circadian responses, and SCN physiology (Feillet et al., 2016; Joye et al., 2023; Kuljis et al., 2013; Pilorz et al., 2020; Rohr et al., 2021; Santhi et al., 2016; Vidafar et al., 2024). These differences may, at least in part, be mediated by ipRGC signaling and should be systematically investigated.

### 6.5 Outlook

Taken together, ipRGCs provide a unique model for understanding how neuronal populations respond to stress in a context-dependent manner. Their dual capacity for resilience and vulnerability highlights the importance of considering not only cell-intrinsic properties but also the temporal and pathological context in which these properties are engaged. Leveraging ipRGC biology for therapeutic and diagnostic purposes will require integrating molecular, physiological, and systems-level insights, with particular attention to subtype diversity and disease-specific mechanisms.

## CRediT authorship contribution statement

**Arianna Cella:** Writing – review & editing, Visualization. **Ludovic S. Mure:** Writing – review & editing, Writing – original draft, Visualization, Supervision, Funding acquisition, Conceptualization.

## AI use

During the preparation of this work the authors used ChatGpt with the prompt “revise, double check syntax and grammar” for each paragraph. After using this tool, the authors reviewed and edited the content as needed and take full responsibility for the content of the publication.

## Declaration of competing interest

The authors declare the following financial interests/personal relationships which may be considered as potential competing interests: Ludovic S Mure reports financial support was provided by Velux Foundation. Ludovic S Mure reports financial support was provided by Fondation pour la recherche contre le Diabete. Ludovic S Mure reports financial support was provided by Novartis Biomedical research foundation. If there are other authors, they declare that they have no known competing financial interests or personal relationships that could have appeared to influence the work reported in this paper.

## Data Availability

No data was used for the research described in the article.
